# Evaluation of the possible association of PDCD-1 and LAG3 gene polymorphisms with hepatocellular carcinoma risk

**DOI:** 10.1186/s12920-023-01526-7

**Published:** 2023-05-02

**Authors:** Jiankai Wei, Zhangxiu Liao, Ying Tao, Shuaiting Liu

**Affiliations:** 1 College of Pharmacy, Youjiang Medical University for Nationalities, Baise, Guangxi China; 2 Basic Medical College, Youjiang Medical University for Nationalities, Baise, Guangxi China; 3 Affiliated Hospital of Youjiang Medical University for Nationalities, Baise, Guangxi China; 4 Guangxi database construction and application engineering research center for intracorporal pharmacochemistry of TCM, Baise, Guangxi China

**Keywords:** Polymorphism, Programmed death-1, Lymphocyte activating 3, Hepatocellular carcinoma, Susceptibility

## Abstract

**Purpose:**

Programmed death-1 (PDCD-1) and lymphocyte activating 3 (LAG3), two important immunosuppressive molecules, play crucial roles in immune escape of tumor cells. This study evaluated the effects of PDCD-1 (rs10204525 and rs36084323), and LAG3 (rs870849 and rs1882545) gene polymorphisms on hepatocellular carcinoma (HCC) risk.

**Methods:**

341 patients with HCC and 350 cancer-free controls in the South Chinese population were included in a population-based case-control study. DNAs were extracted from peripheral blood samples. Genotypes were analyzed using multiplex PCR and sequencing. SNPs were analyzed using multiple inheritance models (co-dominant, dominant, recessive, and over-dominant).

**Results:**

The allele and genotype frequencies of neither of the four polymorphisms, adjusted for age and gender, differed between HCC patients and controls. The differences were also not significant after stratifying by gender and age. According to our results, HCC patients with rs10204525 TC genotype had significantly lower AFP levels than HCC patients with rs10204525 TT genotype (*P* = 0.004). Moreover, the frequency of PDCD-1 rs36084323 CT genotype reduced the risk of TNM grade (CT vs. C/C-T/T: OR = 0.57, 95%CI = 0.37–0.87, *P* = 0.049).

**Conclusion:**

Our results demonstrated that the PDCD-1 (rs10204525 and rs36084323), and LAG3 (rs870849 and rs1882545) polymorphism did not influence the risk of HCC, PDCD-1 rs10204525 TC genotype was associated with the lower AFP levels and rs36084323 CT genotypes were related to HCC tumor grades in the South Chinese samples.

**Supplementary Information:**

The online version contains supplementary material available at 10.1186/s12920-023-01526-7.

## Introduction

Hepatocellular carcinoma (HCC) is the most common cancer of liver cancer and a leading cause of cancer death worldwide. Because of its insidious onset and rapid progression, the diagnosis of HCC is usually delayed rendering treatment difficult. Therefore, screening the risk factor of individuals and diagnosis of HCC at an early stage is supposed to lead to more efficient treatment and better prognosis. The most common risk factors of HCC are viral infections such as hepatitis B virus (HBV) and hepatitis C virus (HCV) infections. However, not all HBV and HCV patients develop HCC during their lifetime. Genetic variation such as single nucleotide polymorphisms (SNPs) has been proved to be an important risk factor for HCC [1]. Studying the relationship between SNPs and HCC susceptibility will shed new light on the theoretical guidance for the clinical diagnosis and prevention of HCC.

HCC suffers from an immunosuppressive environment and T-cell exhaustion, which has been reported to be associated with programmed cell death 1 (PDCD-1) and lymphocyte activating 3 (LAG3), two important coinhibitory regulators of T- cell responses [2, 3]. PDCD-1 is a member of CD28/B7 superfamily and is widely expressed in activated T cells, B cells, and monocytes. As PDCD-1 binds to PD-L1 and PD-L2, it triggers an inhibitory signal against T cell activation and proliferation, as well as cytokine secretion [2]. In patients suffering from HCC, it has been shown that the expression of PDCD-1 on CD8^+^ T cells is constantly increased [4, 5], and tumor evasion and a poor prognosis for HCC were associated with a high frequency of circulating and tumor-infiltrating PDCD-1^+^ CD8^+^ T cells [2, 4, 5].

LAG3 is mainly expressed on the surface of activated natural killer cells and T cells with major histocompatibility complex class II (MHC-II) and fibrinogen-like protein 1 (FGL1) as its ligand. Recent studies found that LAG3 was abundantly expressed in exhausted T cells by using single-cell RNA-seq to examine infiltrating T cells in HCC [6, 7]. Furthermore, antibodies against PD-L1, TIM3, or LAG3 restored responses of HCC-derived T cells to tumor antigens, and combinations of these antibodies had synergistic effects [3].

The above studies suggested that PDCD-1 and LAG3 might play key roles in the pathogenesis of HCC, and genetic variation in PDCD-1 and LAG3 might be related to this process.

The rs36084323 G/A (C/T, PDCD-1.1) and the rs10204525 G/A (C/T, PDCD-1.6) are the two SNP on the chromosome 2 within the PDCD-1 gene that have been studied more frequently. The rs36084323 is located at -606 base pairs upstream of the promoter region at position 242801596 and the rs10204525 G/A (C/T, PDCD-1.6) is located at + 8669 base pairs in the 3’ UTR at position 241,850,169. The relationship between the two SNP and susceptibility to HCC has been reported in several studies [8–11]. However, these conclusions are not always consistent. The SNPs of rs36084323 and rs10204525 were significantly associated with HBV-related cirrhosis and HCC in Chinese Han individuals [8–10]. But no significant association was observed in the gene polymorphisms of rs36084323 and rs10204525 with HCC in the Turkish population [11]. It suggested that ethnicity might play a role in the observed frequencies of PDCD-1 polymorphisms in relation to HCC susceptibility. Therefore, it is needed for studying the relationship between PDCD-1 SNPs and HCC susceptibility in different ethnic populations.

There are few reports about LAG3 gene polymorphism in digestive tract tumors. It has been reported that the polymorphisms of LAG3 rs19922452, rs951818, and rs870849 genes are related to the susceptibility to multiple sclerosis, and rs19922452 TT, rs951818 GG, and rs870849 CT genotypes were significantly associated with decreased risk of multiple sclerosis [12]. So far there is no study on the association between LAG3 genetic polymorphisms and HCC risk.

To the best of our knowledge, data on the relationship between PDCD-1 /LAG3 gene variability and the risk of HCC are lacking for the South Chinese population, and the findings from others cannot be reliably applied to this population due to interpopulation genetic differences. The aim of this study is to test the hypothesis that the polymorphisms of PDCD-1 (rs10204525 and rs36084323), and LAG3 (rs870849 and rs1882545) might be associated with the risk of developing HCC in the South Chinese population, we performed a genotyping analysis in a case-control study. Differences in clinical indicators under different genotypes and the interaction of candidate SNPs in HCC risk were also analyzed.

## Materials and methods

### Study populations

The blood samples were collected from unrelated Chinese individuals (mainly from Guangxi, Yunnan, and Guizhou Province) for this case-control study in the affiliated hospital of Youjiang Medical University for Nationalities between January 2018 and December 2020. In total, the study population comprised 341 patients with HCC and 350 healthy control participants. According to the Chinese Society of Liver Cancer standards, HCC patients were diagnosed based on histopathology or imaging evidence. The inclusion criteria for the control group were the number of neutrophils (range from 1.46*10^9/L^-7.46*10^9/L^) and lymphocytes (range from 0.72*10^9/L^-4.0*10^9^) and the levels of AST (aspartate transaminase, 4–40 U/ml), ALT (alanine transaminase, 12–39 U/ml) and AFP (alpha-fetoprotein, 0.81–13.4 ng/ml) within a normal reference range. In the control group, none of the participants had hepatocellular or other cancers, liver disease by imaging evidence, or a family connection to the experiment group. All control participants tested negative for HBV antigen and anti-HCV antibodies. The age and gender of the HCC patients were matched with those of the control group. Standard clinical laboratory techniques were used to measure AFP levels.

### DNA extraction and SNP analysis

Blood samples were collected into EDTA-containing tubes from participants and stored at -80 °C until analysis. Genomic DNA was isolated from peripheral blood leukocytes using a blood gDNA isolation Kit according to the manufacturer’s instructions (Biomiga, China).

Genotyping was performed by multiplex PCR and sequencing (Sangon, Shanghai, China). The primers used for genotyping were shown in Table 1. The library was prepared with two rounds of PCR. The amplicon products were purified using AMPure XP beads. HiSeq XTen sequencers (Illumina, San Diego, CA) were used for paired-end sequencing, and BWA (version: 0.7.13-r1126) with default parameters was used to map the remaining clean data to the reference genome. Samtools (version: 0.1.18) and Annovar (version: 2018-04-16) were used to calculate each genotype of the target site and detect genetic variants respectively.

**Table 1:** The primers sequence information corresponding to each locus

Gene	locus	Forward	Reverse
PDCD-1	rs10204525	CACTCGGGAGAGGGACATCCTACGG	CCGGCCAACCCCTTTAAATAATTTC
	rs36084323	CGATTAGCCATGGACAGTTGTCATT	TCAACCCCACTCCCATTCTGTCGGA
LAG-3	rs870849	CACCTGTCTTCTCCAAAGGTGAAAG	GCCTCATCCTGTACTTTCTCCATAG
	rs1882545	TGAGCCGTCTACATAAAACAGTTGA	GATAATACATCTCCTGAAGGCCAAT

### GMDR analysis of SNP-SNP interaction

In addition, generalized multifactor dimensionality reduction (GMDR) 0.7 software was used to test for potential interactions (one-to-four-way combinations) among significant SNPs. The GMDR is open-source software and freely available from https://sourceforge.net/projects/gmdr/. The GMDR model showed the distribution of high-risk genotypes and protective genotypes.

### Statistical analysis

Data analysis was performed by SPSS 22.0 statistical package (SPSS, Chicago, IL). The distribution of age between HCC patients and controls was compared by the student’s *t*-test analysis. The chi-squared (χ^2^) test was used to test the deviation from Hardy-Weinberg equilibrium (HWE) for each polymorphism and to assess the differences in the distribution of gender between HCC patients and controls. The association for SNP in PDCD-1 and LAG3 and the risk of HCC was assessed under the inheritance models (codominant, dominant, recessive, and over dominant) using the website for SNP statistics: https://www.snpstats.net. The genotype and allele frequencies of the four SNPs were compared between HCC patients and controls or between TNM I/II and TNM III/IV with the χ^2^ test. The odds ratio (OR) and 95% confidence interval (CI) were estimated using logistic regression analysis. The AFP levels of the patients among groups were compared by Kruskal-Wallis or Mann–Whitney test. A p-value < 0.05 was considered statistically significant.

## Results

### Clinical and demographic characteristics of study populations

Table 2 summarizes the general characteristics of the participants, without significant differences found in regard to age and gender between the HCC and control group. Other demographic variables such as etiology; and clinical features of cases are also outlined in Table 2.

**Table 2:** Demographic and clinical features of patients with HCC and controls

Variables	Patients (*n* = 341)	Controls (*n* = 350)	*P* value
Age (years), mean±SD	51.07 ± 10.86	51.78 ± 8.73	0.346
Gender, n (%)			0.440
Male	300(87.98)	301 (86.00)	
Female	41 (12.02)	49 (14.00)	
AFP (ng/ml)	287.7 (6.26–1210.00)	3.15 (2.47–4.15)	< 0.001
Smoker, n (%)	144 (42.22)		
Alcohol drinkers and HBV positive, n (%)	126 (36.95)		
Alcohol drinkers and HBV negative, n (%)	43 (12.61)		
HBV positive and no alcohol consumption, n (%)	139 (40.76)		
No alcohol consumption and HBV negative, n (%)	33 (9.68)		
TNM stage, n (%)			
I + II	158 (46.33)		
III + IV	183 (53.67)		

HBV, hepatitis B virus; AFP, α-fetoprotein

### Association and stratification analyses of the four candidate SNPs and HCC risk

**Fig. 1:**
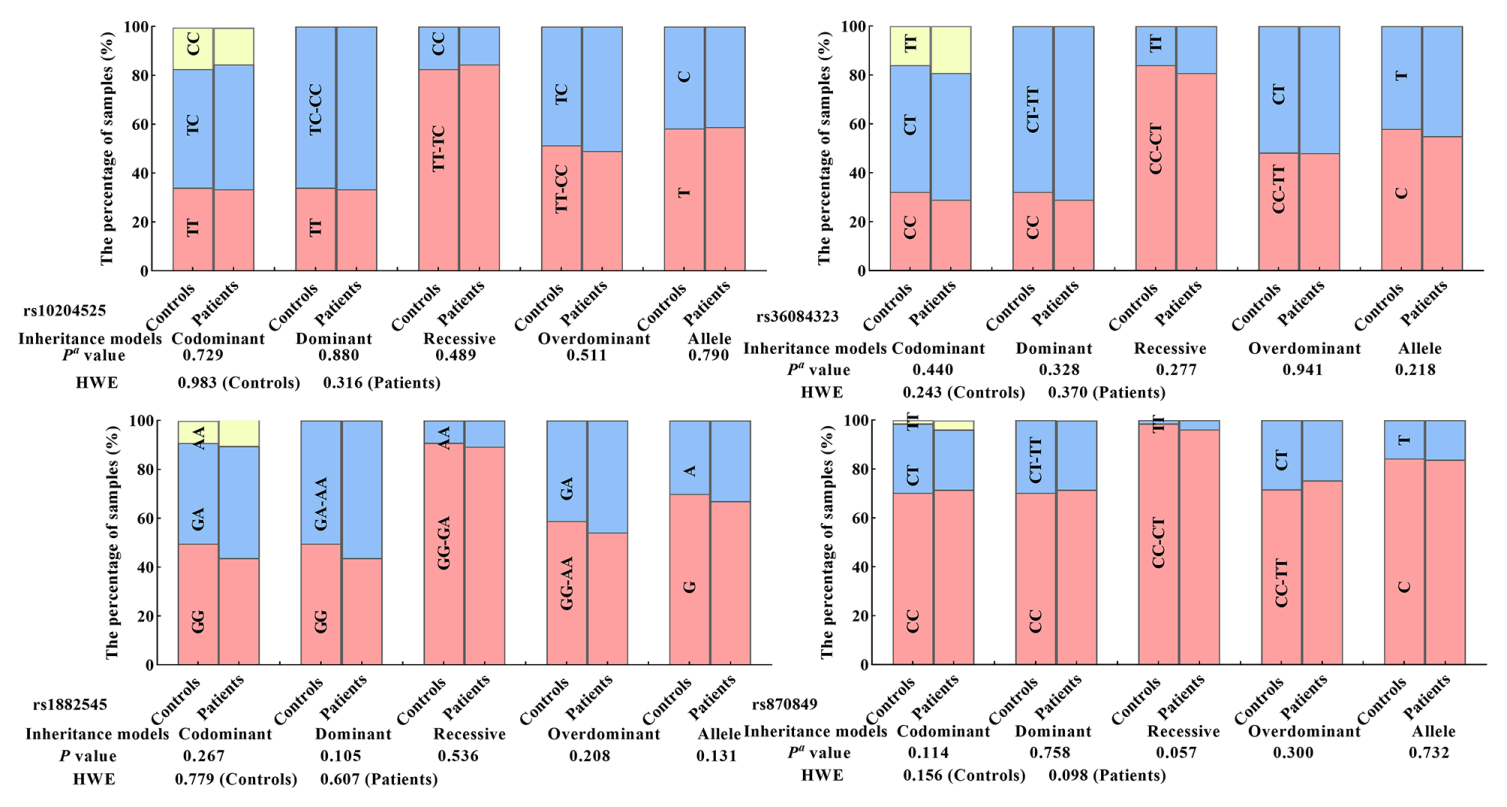
PDCD-1 and LAG3 genotype, genetic model, allele frequency, and HCC susceptibility. (^a^Adjust OR (95% CI) was calculated by logistic regression analysis with adjustments for age and gender)

A total of 691 individuals, including 341 patients with HCC and 350 persons in healthy control, were genotyped for PDCD-1 (rs10204525 and rs36084323), and LAG3 (rs870849 and rs1882545) polymorphisms. The genotype and allele frequencies of the four SNPs in HCC patients and controls were shown in Fig. 1. The number of rs10204525 genotypes was 114 for TT wild type, 174 for TC heterozygous, and 53 for CC mutant homozygous in the HCC and 119 for TT, 170 for TC, and 61 for CC in the controls. The number of rs36084323 genotypes was 99 for CC wild type, 177 for CT heterozygous, and 65 for TT mutant homozygous in the HCC and 113 for CC, 181 for CT, and 56 for TT in the controls. The number of rs18825455 genotypes was 149 for GG wild type, 156 for GA heterozygous, 36 for AA mutant homozygous in the HCC and 174 for GG, 144 for GA, and 32 for AA in the controls. The number of rs870849 genotypes was 244 for CC wild type, 84 for CT heterozygous, and 13 for TT mutant homozygous in the HCC and 246 for CC, 99 for CT, and 5 for TT in the controls. All four SNPs in HCC patients and controls were in HWE (*P* > 0.05), suggesting that there was no population stratification and no sampling bias. Statistical significance was found in none of the four candidate SNPs associated with HCC risk under codominant, dominant, recessive, and over-dominant models (Fig. 1).

**Fig. 2:**
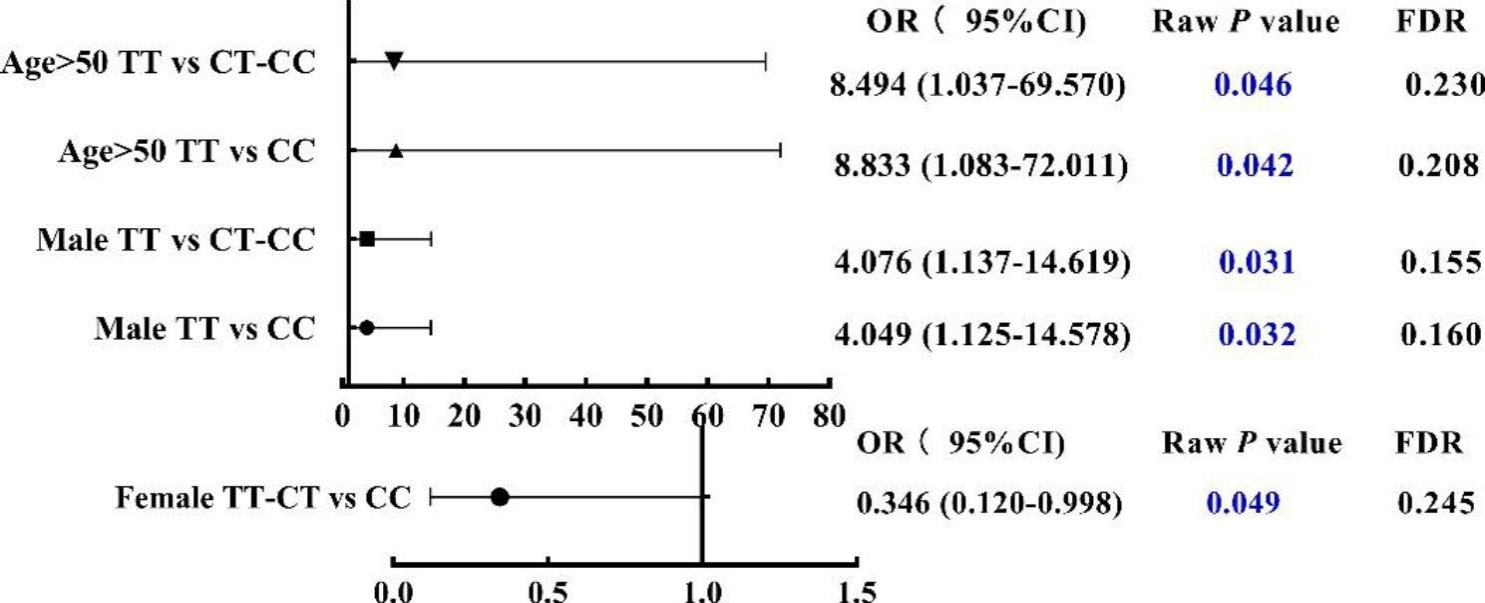
Association analysis of SNP rs870849 with HCC risk after adjusted. OR and P values were adjusted for age and gender by logistic regression

According to the gender and age subgroup analyses (Tables 3 and 4), the raw p values of statistical analysis for the distribution frequency of LAG3 870,849 genotype between HCC and controls were less than 0.05 in participants over 50 years old, in male and female participants under multiple genetic models. However, after Bonferroni correction, there was no statistical significance in none of the four candidate SNPs were associated with HCC risk in all participants (Fig. 2).

**Table 3:** Stratified analyses between PDCD-1 gene polymorphisms and the risk of HCC.

Variable	Genotypes (Patients/controls)			Codominant model		Recessive model	Dominant model
	Wild	Heterozygote	Homozygous				
rs10204525	TT	TC	CC	TC vs. TT	CC vs. TT	CC vs. TC-TT	CC-TC vs. TT
Gender							
Male	99/99	155/151	46/51	1.05 (0.73–1.50)	0.90 (0.55–1.46)	0.87 (0.56–1.35)	1.01 (0.72–1.42)
*P*^*a*^ value				0.80	0.67	0.54	0.96
Female	15/20	19/19	7/10	1.44 (0.56–3.69)	0.98 (0.30–3.22)	0.81 (0.28–2.38)	1.28 (0.54–3.05)
*P*^*a*^ value				0.45	0.98	0.70	0.58
Age							
≤ 50	56/59	83/68	27/31	1.31 (0.79–2.15)	0.92 (0.48–1.76)	0.79 (0.44–1.42)	1.18 (0.74–1.89)
*P*^*a*^ value				0.30	0.80	0.43	0.48
> 50	58/60	91/102	26/30	0.92 (0.58–1.47)	0.93 (0.49–1.76)	0.97 (0.55–1.73)	0.92 (0.59–1.44)
*P*^*a*^ value				0.74	0.82	0.93	0.73
rs36084323	CC	CT	TT	CT vs. CC	TT vs. CC	TT vs. CT-CC	TT-CT vs. CC
Gender							
Male	88/95	157/158	55/48	1.09 (0.76–1.57)	1.24 (0.77–2.02)	1.18 (0.77–1.80)	1.13 (0.80–1.60)
*P*^*a*^ value				0.64	0.38	0.46	0.50
Female	11/18	20/23	10/8	1.40 (0.53–3.67)	1.86 (0.55–6.30)	1.52 (0.53–4.39)	1.52 (0.61–3.77)
*P*^*a*^ value				0.50	0.32	0.44	0.37
Age							
≤ 50	53/52	86/80	27/26	1.09 (0.66–1.80)	1.00 (0.51–1.97)	0.95 (0.52–1.74)	1.07 (0.66–1.72)
*P*^*a*^ value				0.74	1.00	0.86	0.79
> 50	46/61	91/101	38/30	1.14 (0.70–1.84)	1.65 (0.89–3.07)	1.52 (0.89–2.61)	1.26 (0.79–1.98)
*P*^*a*^ value				0.60	0.11	0.12	0.33

^a^Adjust OR (95% CI) was calculated by logistic regression analysis with adjustments for age and gender

**Table 4:** Stratified analyses between LAG3 gene polymorphisms and the risk of HCC

Variable	Genotypes (Patients/controls)			Codominant model		Recessive model	Dominant model
	**Wild**	**Heterozygote**	**Homozygous**				
rs1882545	GG	GA	AA	GA vs. GG	AA vs. GG	AA vs. GA-GG	AA-GA vs. GG
Gender							
Male	135/149	137/124	28/28	1.22 (0.87–1.71)	1.09 (0.62–1.94)	0.99 (0.57–1.72)	1.20 (0.87–1.65)
*P*^*a*^ value				0.25	0.76	0.98	0.27
Female	14/25	19/20	8/4	1.54 (0.61–3.90)	3.77 (0.95–14.95)	3.09 (0.84–11.40)	1.92 (0.81–4.55)
*P*^*a*^ value				0.36	0.06	0.09	0.14
Age							
≤ 50	88/95	157/158	55/48	1.08 (0.67–1.73)	1.30 (0.60–2.81)	1.26 (0.60–2.64)	1.12 (0.71–1.74)
*P*^*a*^ value				0.77	0.51	0.55	0.63
> 50	11/18	20/23	10/8	1.46 (0.95–2.27)	1.42 (0.67–2.99)	1.17 (0.57–2.38)	1.46 (0.96–2.21)
*P*^*a*^ value				0.09	0.36	0.67	0.079
rs870849	CC	CT	TT	CT vs. CC	TT vs. CC	TT vs. CT-CC	TT-CT vs. CC
Gender							
Male	209/213	79/85	12/3	0.98 (0.68–1.41)	4.05 (1.13–14.59)	4.08 (1.14–14.62)	1.09 (0.76–1.54)
*P*^*a*^ value				0.90	0.032	0.031	0.65
Female	35/33	5/14	1/2	0.34 (0.11–1.05)	0.39 (0.03–4.70)	0.48 (0.04–5.77)	0.35 (0.12-1.00)
*P*^*a*^ value				0.06	0.46	0.57	0.049
Age							
≤ 50	126/114	35/40	5/4	0.81 (0.48–1.38)	1.10 (0.27–4.45)	1.16 (0.29–4.66)	0.84 (0.50–1.39)
*P*^*a*^ value				0.44	0.90	0.84	0.49
> 50	118/132	49/59	8/1	0.88 (0.55–1.39)	8.49 (1.04–69.57)	8.83 (1.08–72.01)	1.00 (0.64–1.57)
*P*^*a*^ value				0.57	0.046	0.042	0.99

^a^Adjust OR (95% CI) was calculated by logistic regression analysis with adjustments for age and gender

### Differences in clinical indicators under different genotypes

**Fig. 3:**
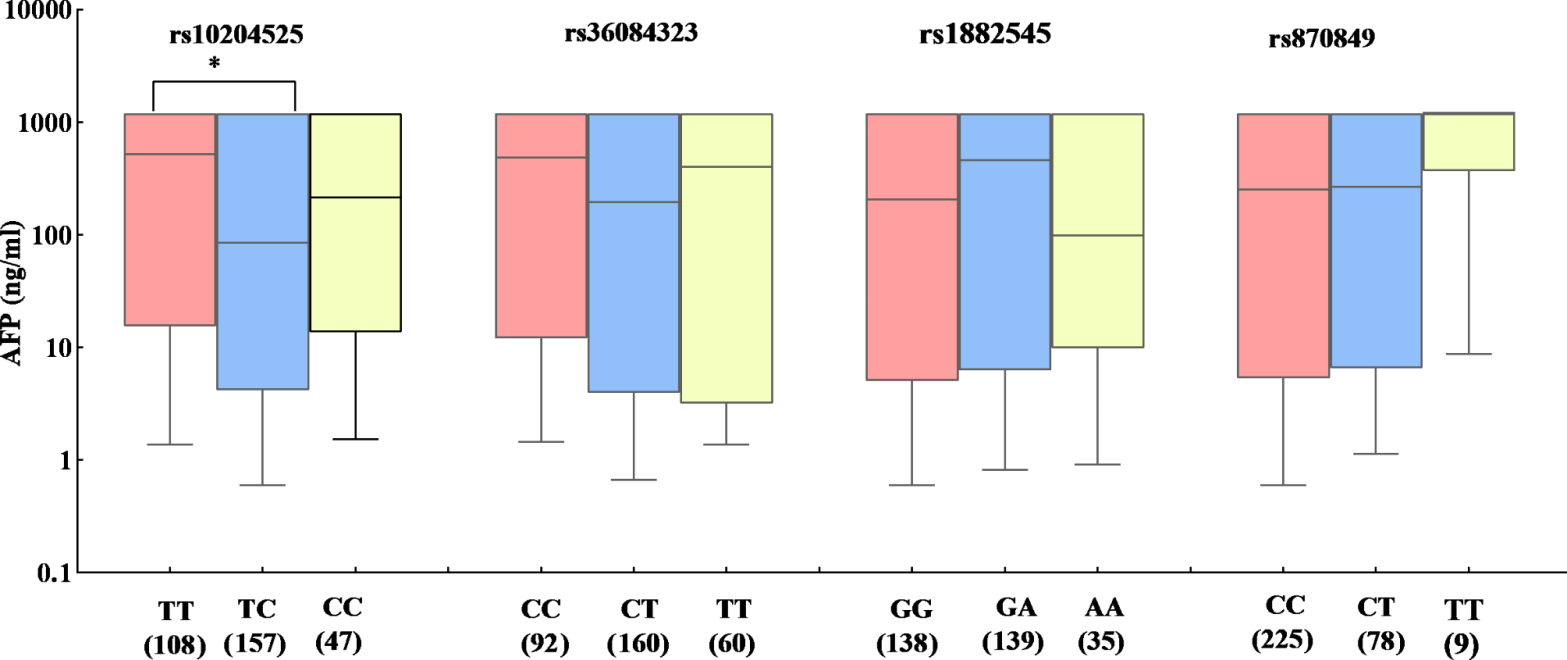
AFP levels of HCC patients based on the genotypes of selected SNPs. (^*^P-values < 0.01)

**Fig. 4:**
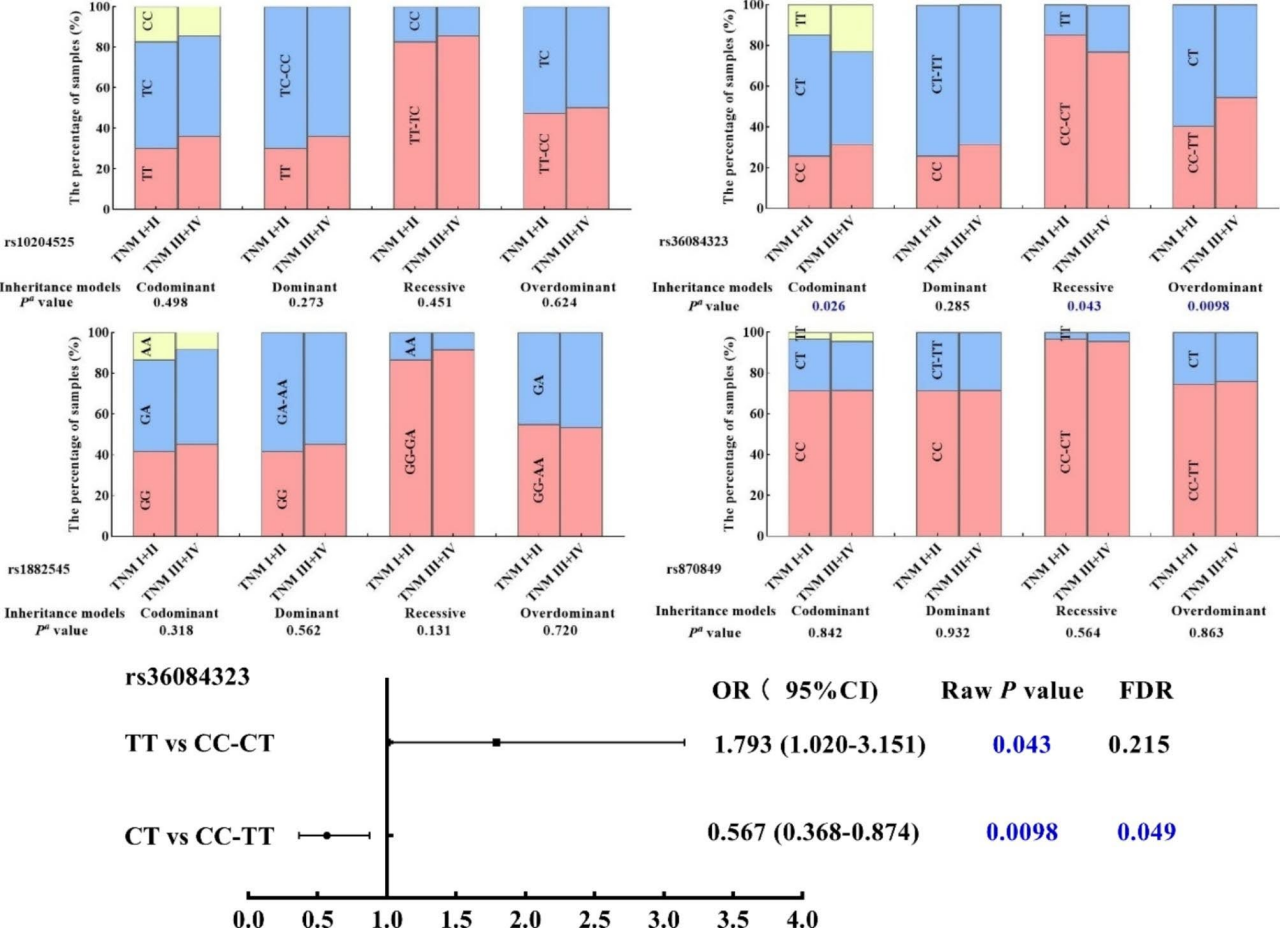
The associated of the different genotypes at rs10204525, rs36084323, rs1882545, and rs870849 with TNM stage. (^a^Logistic regression analyses adjusted for age and gender)

The effects of four candidate SNPs on the level of AFP under different genotypes were also evaluated. The results showed that the level of AFP was significantly lower in HCC patients with rs10204525 TC genotype than that in HCC patients with rs10204525 TT genotype (Mann-Whitney U Test, *P* = 0.004, Fig. 3). Moreover, the differences in genotype frequencies of four candidate SNPs between TNM I/II and TNM III/IV in HCC patients were also analyzed (Fig. 4). The frequency of PDCD-1 rs36084323 CT genotype was lower in HCC TNM III/IV compared to that in HCC TNM I/II and reduced the risk of TNM grade (CT vs. C/C-T/T: OR = 0.57, 95%CI = 0.37–0.87, *P* = 0.049).

### Analysis of GMDR

**Fig. 5:**
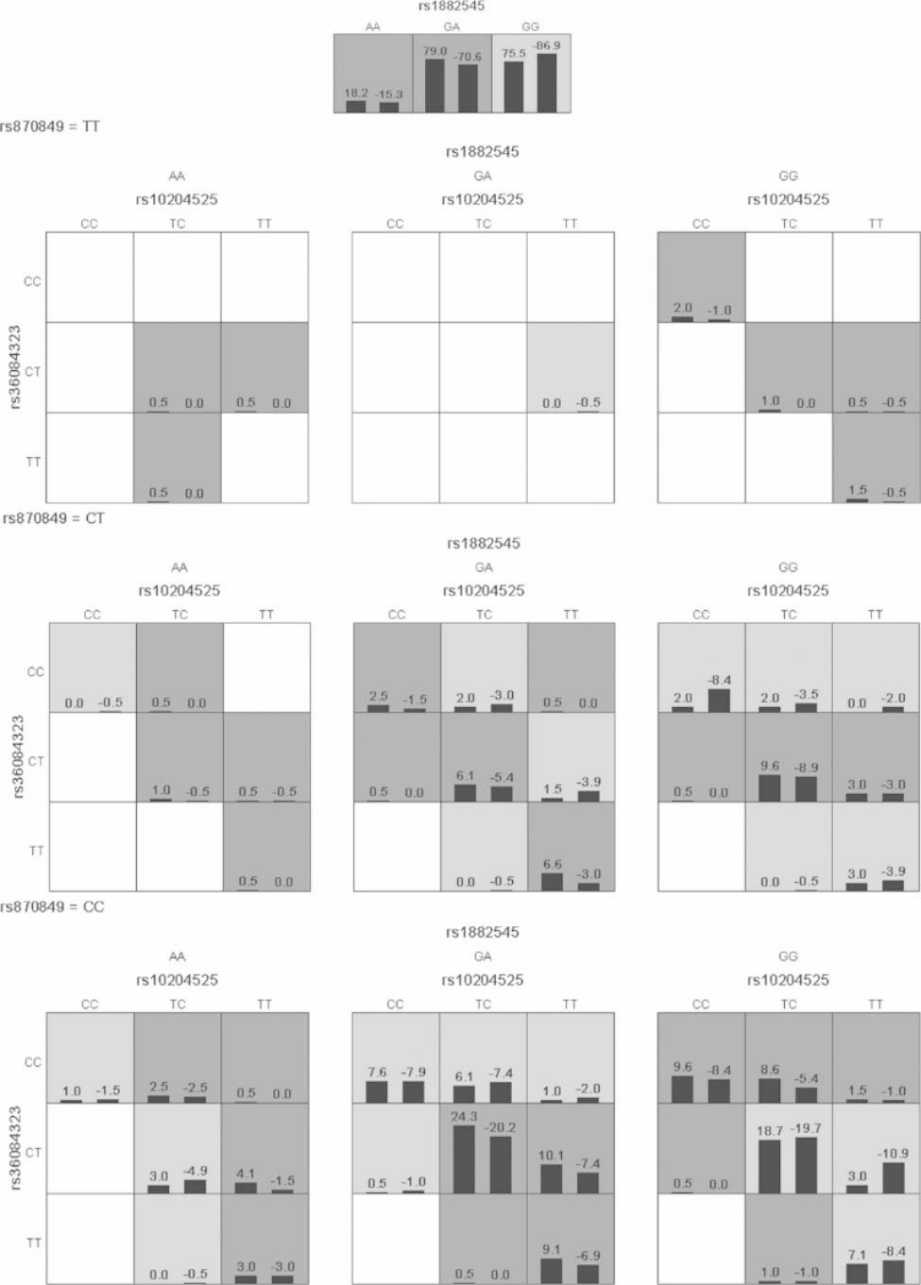
GMDR analysis of PDCD-1 (rs10204525, rs36084323), and LAG-3 (rs870849, rs1882545) interaction

The interaction of candidate SNPs in HCC risk was analyzed and evaluated using GMDR (Table 5). The best two-site model for predicting the HCC risk is rs1882545 (testing accuracy = 0.533, cross-validation consistency = 10/10, *P* = 0.055); the four-site model is rs10204525, rs36084323, rs1882545, rs870849 (testing accuracy = 0.515, cross-validation consistency = 10/10, *P* = 0.172). Figure 5 showed the interaction of “SNP-SNP” in different loci model combinations. The light gray lattice indicated a low risk of HCC, the dark gray lattice indicated a high risk, and no color-filled lattice signified no data. Although the cross-validation consistency was higher, it was unable to reach statistical significance. It suggested that no significant interactions between PDCD-1 and LAG3 polymorphisms affected the susceptibility of HCC.

**Table 5:** SNP-SNP interaction models analyzed by the GMDR method

Model	Training Bal.Acc	Testing Bal.Acc	P	CVC
rs1882545	0.533	0.533	0.055	10/10
rs1882545 rs870849	0.546	0.501	0.377	5/10
rs36084323 rs1882545 rs870849	0.576	0.513	0.377	7/10
rs10204525 rs36084323 rs1882545 rs870849	0.586	0.515	0.172	10/10

Bal. Acc., balanced accuracy; CVC, cross-validation consistency

In each box, the left bar represents HCC patients and the right bar represents controls. The light gray lattice indicates a low risk of HCC and the dark gray lattice indicates a high risk of HCC; the empty lattice means no data.

## Discussion

As a result of chronic infection with hepatitis B and C viruses, aflatoxin-contaminated food, heavy alcohol consumption, obesity, diabetes type 2, and smoking, the risk for liver cancer is high [13]. The key risk factors vary from region to region. In the present study, among 341 HCC patients randomly collected from Youjiang Medical University for Nationalities in South China, HBV infection comprised 77.71% of cases, suggesting HBV was the major risk factor for HCC patients. The mean age of patients with HCC was 51.07 years old, and slightly younger than that in other studies [14].

Genetic polymorphisms involved in the immune response are already known to be related to the anti-tumor immune response and influence the occurrence of HCC [15]. PDCD-1 and LAG3, two immunosuppressive molecules, play important roles in tumor-cell-mediated immune escape. We evaluated the effect of PDCD-1 and LAG3 gene polymorphisms on the risk of hepatocellular carcinoma (HCC) in this study. Our results indicated there were no associations between PDCD-1 rs10204525, PDCD-1 rs36084323, and LAG3 rs1882545, rs870849 polymorphisms and the risk of HCC in our population.

Based on the inhibitory role of LAG3 in anti-tumor responses, several studies have focused on the association between LAG3 rs3782735A/G genetic polymorphisms and cancer risk development [15–18]. Rs3782735A/G are intronic variants of LAG-3 gene. It is found to have potential associations with multiple myeloma (MM) risk in women and AA genotype reduced the risk MM (OR = 0.69) [16]. Another research has previously reported increased survival rates among patients with locoregional gastric cancer carrying LAG3 rs3782735 AG and GG genotypes in the Japanese cohort [17]. Stremitzer and colleagues found in Australian patients with resected colorectal liver metastases harboring LAG3 rs3782735 GG genotype separated patients with intermediate risk for death [18]. These results suggest that LAG3 gene polymorphism may play a role in the pathogenesis and development of cancer. However, the association between LAG3 genetic polymorphisms and HCC risk remains unclear.

There is a non-synonymous SNP in LAG3 rs870849 T/C which causes an amino acid substitution from isoleucine to threonine at position 455 in exon 7 [19]. So, LAG3 rs870849 genetic variants may affect both the expression and structure of LAG3 and the occurrence or progression of cancer. But there is little research on the association between LAG3 rs870849 and the risk of cancer. Frequencies of LAG-3 rs870849 CC, CT, and TT genotypes were 246 (70.3%), 99 (28.3%), and 5 (1.4%) in controls in our study, which are similar to those in Chinese Dai in Xishuangbanna (CDX) (*P* = 0.72), Han Chinese in Beijing, China (CHB) (*P* = 0.16) and Southern Han Chinese, China (CHS) (*P* = 0.30) population (http://asia.ensembl.org/Homo_sapiens/Variation/Population?db=core;r=12:6777354-6778354;v=rs870849;vdb=variation;vf=729067871). In our study we found a slight increase of the LAG3 rs870849 TT genotype in HCC patients (3.8%) with compared to the controls (1.4%). The distribution frequencies of LAG3 rs870849 TT genotypes in male patients (4.0%) and old than 50 years HCC patients (4.6%) were also increased compared with their controls (1.0% and 0.5%), respectively. However, the number of LAG3 rs870849 TT genotype was too few, the increase was not statistically significant. Thus, an increasing sample size is required for further studies on the association between LAG3 rs870849 and the risk of HCC.

Rs1882545 G/A is located on the intron of the LAG3 human gene. As far as we know, there is very little literature evaluating the association between LAG-3 rs1882545 genetic polymorphisms and disease risk development. Frequencies of LAG-3 rs1882545 GG, GA, and AA genotypes were 174 (49.7%), 144 (44.1%), and 32 (9.1%) in controls, which are similar to those in CDX (*P* = 0.51), CHS (*P* = 0.75) and Kinh in Ho Chi Minh City in Vietnam (KHV) (*P* = 0.97) population (http://asia.ensembl.org/Homo_sapiens/Variation/Population?db=core;r=12:6775744-6776744;v=rs1882545;vdb=variation;vf=729509994). In our study we found a slight but not statistically significant increase of the LAG3 rs1882545 A allele in HCC patients (33.4%) with compared to the controls (29.7%). Moreover, the LAG-3 rs1882545 A allele was more common in females with HCC (35/82; 42.7%) compared to female controls (28/98; 28.6%) in our study, but the increase was not statistically significant. Larger multicenter studies and increasing sample sizes are required to further study.

Although we did not find a relationship between LAG3 gene polymorphism and the risk of HCC in our study, possibly due to the small sample size, this is the first study to investigate LAG3 gene polymorphism in HCC. This will provide a foundation for further research on the impact of LAG3 gene polymorphism on other biological functions of HCC, such as prognosis or response to immune checkpoint antibodies.

Previous studies showed that PDCD-1 was highly expressed in tumor tissues in patients with HBV-related HCC and genotype AA (TT) of PD1 rs10204525 was associated with significantly increased PDCD-1 expression [20, 21]. It was suggested that rs10204525 polymorphism might influence the expression of PDCD-1 and hence might affect the development of HCC. Li Z et al. found PDCD-1 rs10204525 AA genotype was independently associated with HBV-related HCC compared with patients without cirrhosis and HCC (patients with asymptomatic carriers and chronic hepatitis) in Northwest China [8]. Another research has previously reported PDCD-1 rs10204525 C > T SNP acquired significance after adjusting for other risks, being most notable in the smaller numbers of women with NAFLD-HCC in Caucasian patients [22]. Contrary to these studies, the rs10204525 SNP was found to have an insignificant role in patients with HCV-related hepatic disorders (chronic infection, cirrhosis, and HCC) in the Italian cohort [23] and was not a predisposing factor for HCC development in the Turkish population [11]. In the present study, our results also demonstrated no association between rs10204525 T > C and HCC risk, which was consistent with De Re V’s study in the Italian cohort and Demirci AF’s study in the Turkish population [11, 23]. Although the allele distribution of the PDCD-1 rs10204525 genotype in the control group in this study was not similar to the distribution of the previously studied Northwest China and other population, it had no differences with the distribution of CDX and KHV populations (http://asia.ensembl.org/Homo_sapiens/Variation/Population?db=core;v=rs10204525;vdb=variation, *P* = 0.89 and *P* = 0.78, respectively). Interestingly, our results suggested that HCC patients carrying the rs10204525 TC genotype were more likely to have a lower AFP level. Serum AFP level is a useful predictor of the outcome of HCC in patients, and higher level of AFP is associated with poor prognosis of HCC in patients receiving TACE (transcatheter arterial chemoembolization) [24]. These findings shed light on genetic determinants of serum AFP level and provide a more comprehensive understanding that PDCD-1 rs10204525 SNP may have an impact on the prognosis of HCC patients. These results could suggest the need for a further inquiry into the mechanism of how PDCD-1 rs10204525 is associated with AFP in HCC and the effect of rs10204525 on the prognosis of HCC patients.

In the promoter region of the PDCD-1 gene, there is the rs36084323 A/G (T/C) SNP. It is demonstrated that the promoter activity was significantly higher in the construct with G allele than in that with the A allele and higher promoter activity caused by rs36084323 G (C) allele may have an influence on gene expression and susceptibility to disease [25]. A meta-analysis shows the PDCD-1 rs36084323 SNP is associated with decreased cancer risk (GG + GA vs. AA, OR = 0.903, 95% CI = 0.819–0.995, *P* = 0.038) [26]. The association between rs36084323 polymorphism and the risk of HCC was observed in several studies [10, 11, 23]. However, the results of the studies are conflicting. In Peng’s study, as compared to patients without HCC (asymptomatic carriers, chronic hepatitis, and cirrhosis), the genotype GA of PDCD-1 rs36084323 was independently associated with HCC in Chinese patients (*P* = 0.024, OR = 0.459) [9]. Nevertheless, the allele frequency distribution in the Caucasian population all samples were GG homozygous and there were no differences between HCV-associated HCC patients and controls [23]. Another study also found PDCD-1.1 AA genotype was not observed in both the patients with HCC and the control group, and the frequencies of GG and GA genotypes were very similar between patients with HCC patients and controls [11]. Our study also showed no significant relationship between this SNP and HCC in the South Chinese population. In the present study, CC (GG), CT (GA), and TT (AA) genotype frequencies in controls were 32.3%, 51.7%, and 16.0% respectively were similar to levels in CHB, CHS, Japanese in Tokyo, Japan (JPT), and KHV (http://asia.ensembl.org/Homo_sapiens/Variation/Population?db=core;v=rs36084323;vdb=variation). It is worth noting, our results indicated that HCC patients carrying the PDCD-1 rs36084323 CT genotype were significantly associated with lower TNM stage compared with those patients carrying the homozygote genotype. Previous study showed mutant PDCD-1 rs36084323 T allele had significantly lower PDCD1 transcriptional activity [27]. It suggested PDCD-1 rs36084323 CT genotype might have an effect on the expression and immunosuppressive function of PDCD-1. TNM stage is a better predictor of cancer prognosis, and advanced TNM stage is associated with poor prognosis of HCC in patients receiving TACE [24]. In our study, the relationship of PDCD-1 rs36084323 CT genotype with TNM stage might be suggested PDCD-1 rs36084323 SNP was associated with the prognosis of HCC, but not associated with the occurrence of HCC. Further studies are required to fully elucidate the relationship of the prognosis in HCC patients with the PDCD-1 rs36084323 CT genotype.

PDCD-1/PD-L1 pathway blockade is a promising treatment strategy in HCC. However, a significant number of patients with HCC do not respond to anti-programmed cell death 1 (PD1) therapies. Macek Jilkova Z and colleagues observed a trend of LAG3 upregulation on circulating T cells in nonresponding patients to PDCD-1/PD-L1 pathway blockade [28]. It suggests that the PDCD-1/PD-L1 pathway and LAG3 pathway might play a synergistic role in HCC development. Therefore, the interaction of PDCD-1 and LAG3 SNPs in HCC risk among participants was analyzed and evaluated in our study. But no significant interaction between PD1 and LAG3 polymorphisms was found to affect the susceptibility of HCC.

Nevertheless, there are some limitations to this study. First, the results were obtained mainly in Chinese populations from Guangxi, Yunnan, and Guizhou Provinces and, thus, may not be applicable to other populations. PDCD-1 and LAG3 frequencies may vary by ethnicity, which requires further research. Second, although we found there was no difference in the genotype distribution of PDCD-1 (rs10204525 and rs36084323), and LAG3 (rs870849 and rs1882545) among different risk factors such as HBV infection and alcohol intake in HCC patients (data not shown), the addition of intermediate control groups bearing the same environmental risk factors such as long-term cirrhotic HBV, drinking and smoking status could add value to our study. Third, if the impacts of different genotypes of PDCD-1 and LAG3 on the expression and immunosuppressive function will be further observed, it will better explain the effect of genetic polymorphism of PDCD-1 and LAG3 on the biological function of HCC. Finally, our analysis was limited by the size of the sample of HCC patients, especially for females, which may have reduced its statistical power.

This study found no link between four polymorphisms in the PDCD-1 gene, rs10204525 T/C, rs36084323 C/T, LAG3 gene, rs870849 C/T, rs1882545 G/A, and HCC risk. However, it was found that the rs10204525 TC genotype in HCC patients was associated with the AFP level and rs36084323 CT genotypes were related to the HCC tumor TNM stage. In light of the roles of AFP levels and TNM staging in the prognosis of HCC, it is necessary to test the relationship between PDCD1 rs10204525 and rs36084323 SNPs and the prognosis of HCC in the future study. Additionally, due to the small sample size, some unavoidable limitations were present. Thus, further studies, including patients of different ethnic origins, are required to validate these findings in a significantly larger series in order to clarify the exact relationship between these SNPs and HCC development.

## Electronic supplementary material

Below is the link to the electronic supplementary material.

Supplementary Material 1

## Data Availability

The data that support the findings of this study are available on request from the corresponding author.

## References

[1] Jiang J, Chen HN, Jin P, Zhou L, Peng L, Huang Z, Qin S, Li B, Ming H, Luo M, Xie N, Gao W, Nice EC, Yu Q, Huang C (2023). Targeting PSAT1 to mitigate metastasis in tumors with p53-72Pro variant. Signal Transduct Target Ther.

[2] Shi F, Shi M, Zeng Z, Qi RZ, Liu ZW, Zhang JY, Yang YP, Tien P, Wang FS (2011). PD-1 and PD-L1 upregulation promotes CD8(+) T-cell apoptosis and postoperative recurrence in hepatocellular carcinoma patients. Int J Cancer.

[3] Zhou G, Sprengers D, Boor PPC, Doukas M, Schutz H, Mancham S, Pedroza-Gonzalez A, Polak WG, de Jonge J, Gaspersz M, Dong H, Thielemans K, Pan Q, IJzermans JNM, Bruno MJ, Kwekkeboom J (2017). Antibodies against Immune Checkpoint Molecules restore functions of Tumor-Infiltrating T cells in Hepatocellular Carcinomas. Gastroenterology.

[4] Barsch M, Salié H, Schlaak AE, Zhang Z, Hess M, Mayer LS, Tauber C, Otto-Mora P, Ohtani T, Nilsson T (2022). T-cell exhaustion and residency dynamics inform clinical outcomes in hepatocellular carcinoma. J Hepatol.

[5] Dai X, Xue J, Hu J, Yang SL, Chen GG, Lai PBS, Yu C, Zeng C, Fang X, Pan X, Zhang T (2017). Positive expression of programmed death ligand 1 in Peritumoral Liver tissue is Associated with poor survival after curative resection of Hepatocellular Carcinoma. Transl Oncol.

[6] Zheng C, Zheng L, Yoo JK, Guo H, Zhang Y, Guo X, Kang B, Hu R, Huang JY, Zhang Q (2017). Landscape of infiltrating T cells in Liver Cancer revealed by single-cell sequencing. Cell.

[7] Liu F, Liu W, Sanin DE, Jia G, Tian M, Wang H, Zhu B, Lu Y, Qiao T, Wang X (2020). Heterogeneity of exhausted T cells in the tumor microenvironment is linked to patient survival following resection in hepatocellular carcinoma. Oncoimmunology.

[8] Li Z, Li N, Zhu Q, Zhang G, Han Q, Zhang P, Xun M, Wang Y, Zeng X, Yang C, Liu Z (2013). Genetic variations of PD1 and TIM3 are differentially and interactively associated with the development of cirrhosis and HCC in patients with chronic HBV infection. Infect Genet Evol.

[9] Zhang G, Liu Z, Duan S, Han Q, Li Z, Lv Y, Chen J, Lou S, Li N (2010). Association of polymorphisms of programmed cell death-1 gene with chronic hepatitis B virus infection. Hum Immunol.

[10] Peng H, Li QL, Hou SH, Hu J, Fan JH, Guo JJ (2015). Association of genetic polymorphisms in CD8 + T cell inhibitory genes and susceptibility to and progression of chronic HBV infection. Infect Genet Evol.

[11] Demirci AF, Demirtas CO, Eren F, Yilmaz D, Keklikkiran C, Ozdogan OC, Gunduz F (2020). Evaluation of the association between programmed cell Death-1 gene polymorphisms and Hepatocellular Carcinoma susceptibility in turkish subjects. A pilot study. J Gastrointestin Liver Dis.

[12] Zhang Z, Duvefelt K, Svensson F, Masterman T, Jonasdottir G, Salter H, Emahazion T, Hellgren D, Falk G, Olsson T (2005). Two genes encoding immune-regulatory molecules (LAG3 and IL7R) confer susceptibility to multiple sclerosis. Genes Immun.

[13] Thomas London W, Petrick JL, McGlynn KA. Liver cancer. In: Thun M, Linet MS, Cerhan JR, Haiman CA, Schottenfeld D, editors. Cancer Epidemiology and Prevention. 4th ed. Oxford University Press; 2018. pp. 635–60.

[14] Chao X, Feng X, Shi H, Wang Y, Wang L, Shen H, Zha Q, Chen Y, Jiang C (2020). MIR17HG polymorphism (rs7318578) is associated with liver cancer risk in the chinese Han population. Biosci Rep.

[15] Wagner M, Jasek M, Karabon L (2021). Immune Checkpoint Molecules-Inherited variations as markers for Cancer Risk. Front Immunol.

[16] Lee KM, Baris D, Zhang Y, Hosgood HD, Menashe I, Yeager M, Zahm SH, Wang SS, Purdue MP, Chanock S (2010). Common single nucleotide polymorphisms in immunoregulatory genes and multiple myeloma risk among women in Connecticut. Am J Hematol.

[17] Sunakawa Y, Cao S, Volz NB, Berger MD, Yang D, Parekh A, Zhang W, Matsusaka S, Ning Y, Stremitzer S (2017). Genetic variations in immunomodulatory pathways to predict survival in patients with locoregional gastric cancer. Pharmacogenomics J.

[18] Stremitzer S, Sunakawa Y, Zhang W, Yang D, Ning Y, Stintzing S, Sebio A, Yamauchi S, Matsusaka S, El-Khoueiry R (2015). Variations in genes involved in immune response checkpoints and association with outcomes in patients with resected colorectal liver metastases. Pharmacogenomics J.

[19] Wang S, Zhang X, Leng S, Xu Q, Sheng Z, Zhang Y, Yu J, Feng Q, Hou M, Peng J, Hu X (2021). Immune Checkpoint-Related gene polymorphisms are Associated with Primary Immune Thrombocytopenia. Front Immunol.

[20] Li Z, Li N, Li F, Zhou Z, Sang J, Chen Y, Han Q, Lv Y, Liu Z (2016). Immune checkpoint proteins PD-1 and TIM-3 are both highly expressed in liver tissues and correlate with their gene polymorphisms in patients with HBV-related hepatocellular carcinoma. Med (Baltim).

[21] Zhang G, Li N, Zhang P, Li F, Yang C, Zhu Q, Han Q, Lv Y, Zhou Z, Liu Z (2014). PD-1 mRNA expression is associated with clinical and viral profile and PD1 3’-untranslated region polymorphism in patients with chronic HBV infection. Immunol Lett.

[22] Eldafashi N, Darlay R, Shukla R, McCain MV, Watson R, Liu YL, McStraw N, Fathy M, Fawzy MA, Zaki MYW (2021). A PDCD1 role in the genetic predisposition to NAFLD-HCC?. Cancers (Basel).

[23] De Re V, Tornesello ML, De Zorzi M, Caggiari L, Pezzuto F, Leone P, Racanelli V, Lauletta G, Gragnani L, Buonadonna A (2019). Clinical significance of polymorphisms in Immune Response genes in Hepatitis C-Related Hepatocellular Carcinoma. Front Microbiol.

[24] Wang XC, Wang F, Quan QQ. Roles of XRCC1/XPD/ERCC1 polymorphisms in Predicting Prognosis of Hepatocellular Carcinoma in Patients receiving transcatheter arterial chemoembolization. Genet Test Mol Biomarkers. 2016 Apr;20(4):176–84. 10.1089/gtmb.2015.0267. Epub 2016 Feb 26. PMID: 26918371. 10.1089/gtmb.2015.0267 26918371

[25] Ishizaki Y, Yukaya N, Kusuhara K, Kira R, Torisu H, Ihara K, Sakai Y, Sanefuji M, Pipo-Deveza JR, Silao CL (2010). PD1 as a common candidate susceptibility gene of subacute sclerosing panencephalitis. Hum Genet.

[26] Zhang W, Song Y, Zhang X (2021). Relationship of programmed Death-1 (PD-1) and programmed death Ligand-1 (PD-L1) polymorphisms with overall Cancer susceptibility: an updated Meta-analysis of 28 studies with 60 612 subjects. Med Sci Monit.

[27] Chen DP, Wen YH, Wang WT, Lin WT. Exploring the Bio-Functional Effect of single nucleotide polymorphisms in the promoter region of the TNFSF4, CD28, and PDCD1 genes. J Clin Med. 2023 Mar;10(6):2157. 10.3390/jcm12062157. PMID: 36983159; PMCID: PMC10058121. 10.3390/jcm12062157 PMC10058121 36983159

[28] Macek Jilkova Z, Aspord C, Kurma K, Granon A, Sengel C, Sturm N, Marche PN, Decaens T. Immunologic Features of Patients With Advanced Hepatocellular Carcinoma Before and During Sorafenib or Anti-programmed Death-1/Programmed Death-L1 Treatment. Clin Transl Gastroenterol. 2019;10(7): e00058.Zhang G (2010). Association of polymorphisms of programmed cell death-1 gene with chronic hepatitis B virus infection. Hum Immunol 71(12):1209–1213. 10.14309/ctg.0000000000000058 PMC6708670 31295151

