# Supplementary material for: Evaluation of the possible association of PDCD-1 and LAG3 gene polymorphisms with hepatocellular carcinoma risk
Source: BMC Med Genomics. 2023 May 2;16:92. doi: 10.1186/s12920-023-01526-7 (PMC10155321; doi:10.1186/s12920-023-01526-7)
Supplement: Supplementary file 1 — Supplementary Material 1 [file 12920_2023_1526_MOESM1_ESM.docx]

Table 1 PDCD-1 genotype, genetic model, allele frequency, and HCC susceptibility

| SNP | Genotype | Patients (n, %) | Controls (n, %) | *OR* (95%*CI*) | *P* value ^a^ |
| --- | --- | --- | --- | --- | --- |
| rs10204525 |  |  |  |  |  |
| Allele | T | 402 (58.9) | 408 (58.3) | 1.00 | 0.79 |
|  | C | 280 (41.1) | 292 (41.7) | 0.97 (0.78-1.20) |  |
| Codominant | TT | 114 (33.4) | 119 (34.0) | 1.00 | 0.73 |
|  | TC | 174 (51.1) | 170 (48.6) | 1.07 (0.77-1.49) |  |
|  | CC | 53 (15.5) | 61 (17.4) | 0.90 (0.58-1.41) |  |
| Dominant | TT | 114 (33.4) | 119 (34.0) | 1.00 | 0.88 |
|  | TC-CC | 227 (66.6) | 231 (66.0) | 1.03 (0.75-1.41) |  |
| Recessive | TT-CT | 288 (84.5) | 289 (82.6) | 1.00 | 0.49 |
|  | CC | 53 (15.5) | 61 (17.4) | 0.87 (0.58-1.30) |  |
| Overdominant | TT-CC | 167 (49.0) | 180 (51.4) | 1.00 | 0.51 |
|  | TC | 174 (51.0) | 170 (48.6) | 1.11 (0.82-1.49) |  |
| rs36084323 |  |  |  |  |  |
| Allele | C | 375 (55.0) | 407 (58.1) | 1.00 | 0.22 |
|  | T | 307 (45.0) | 293 (41.9) | 1.14 (0.92-1.42) |  |
| Codominant | CC | 99 (29.0) | 113 (32.3) | 1.00 | 0.44 |
|  | CT | 177 (51.9) | 181 (51.7) | 1.13 (0.80-1.58) |  |
|  | TT | 65 (19.1) | 56 (16.0) | 1.34 (0.86-2.10) |  |
| Dominant | CC | 99 (29.0) | 113 (32.3) | 1.00 | 0.33 |
|  | CT-TT | 242 (71.0) | 237 (67.7) | 1.18 (0.85-1.63) |  |
| Recessive | CC-CT | 276 (80.9) | 294 (84.0) | 1.00 | 0.28 |
|  | TT | 65 (19.1) | 56 (16.0) | 1.24 (0.84-1.85) |  |
| Overdominant | CC-TT | 164 (48.1) | 169 (48.3) | 1.00 | 0.94 |
|  | CT | 177 (51.9) | 181 (51.7) | 1.01 (0.75-1.36) |  |

^a^Adjust OR (95% CI) were calculated by logistic regression analysis with adjustments for age and gender.

Table 2 LAG3 genotype, genetic model, allele frequency, and HCC susceptibility

| SNP | Genotype | Patients (n, %) | Controls (n, %) | *OR* (95%*CI*) | *P* value ^a^ |
| --- | --- | --- | --- | --- | --- |
| rs1882545 |  |  |  |  |  |
| Allele | G | 454 (66.6) | 492 (70.3) | 1.00 | 0.13 |
|  | A | 228 (33.4) | 208 (29.7) | 1.19 (0.95-1.50) |  |
| Codominant | GG | 149 (43.7) | 174 (49.7) | 1.00 | 0.27 |
|  | GA | 156 (45.7) | 144 (41.1) | 1.27 (0.93-1.75) |  |
|  | AA | 36 (10.6) | 32 (9.1) | 1.32 (0.78-2.23) |  |
| Dominant | GG | 149 (43.7) | 174 (49.7) | 1.00 | 0.11 |
|  | GA-AA | 192 (56.3) | 176 (50.3) | 1.28 (0.95-1.73) |  |
| Recessive | GG-GA | 305 (89.4) | 318 (90.9) | 1.00 | 0.54 |
|  | AA | 36 (10.6) | 32 (9.1) | 1.17 (0.71-1.94) |  |
| Overdominant | GG-AA | 185 (54.2) | 206 (58.9) | 1.00 | 0.21 |
|  | G/A | 156 (45.8) | 144 (41.1) | 1.21 (0.90-1.64) |  |
| rs870849 |  |  |  |  |  |
| Allele | C | 572 (83.9) | 591 (84.4) | 1.00 | 0.73 |
|  | T | 110 (16.1) | 109 (15.6) | 1.05 (0.79-1.41) |  |
| Codominant | CC | 244 (71.6) | 246 (70.3) | 1.00 | 0.11 |
|  | CT | 84 (24.6 | 99 (28.3) | 0.86 (0.61-1.22) |  |
|  | TT | 13 (3.8) | 5 (1.4) | 2.65 (0.93-7.57) |  |
| Dominant | CC | 244 (71.6) | 246 (70.3) | 1.00 | 0.76 |
|  | CT-TT | 97 (28.4) | 104 (29.7) | 0.95 (0.68-1.32) |  |
| Recessive | CC-TC | 328 (96.2) | 345 (98.6) | 1.00 | 0.057 |
|  | TT | 13 (3.8) | 5 (1.4) | 2.76 (0.97-7.85) |  |
| Overdominant | CC-TT | 257 (75.4) | 251 (71.7) | 1.00 | 0.30 |
|  | CT | 84 (24.6) | 99 (28.3) | 0.84 (0.59-1.18) |  |

^a^Adjust OR (95% CI) were calculated by logistic regression analysis with adjustments for age and gender

Table 3 The associated of the different genotypes at rs10204525, rs36084323, rs1882545, and rs870849 with TNM stage

| SNP | Genotype | TNM III/IV (n, %) | TNM I/II (n, %) | *OR* (95%*CI*) | *P* value ^a^ |
| --- | --- | --- | --- | --- | --- |
| rs10204525 |  |  |  |  |  |
| Codominant | TT | 66 (36.1) | 48 (30.4) | 1.00 | 0.50 |
|  | TC | 91 (49.7) | 83 (52.5) | 0.80 (0.50-1.29) |  |
|  | CC | 26 (14.2) | 27 (17.1) | 0.70 (0.36-1.34) |  |
| Dominant | TT | 66 (36.1) | 48 (30.4) | 1.00 | 0.27 |
|  | TC-CC | 117 (63.9) | 110 (69.6) | 0.78 (0.49-1.22) |  |
| Recessive | TT-TC | 157 (85.8) | 131 (82.9) | 1.00 | 0.45 |
|  | CC | 26 (14.2) | 27 (17.1) | 0.80 (0.44-1.44) |  |
| Overdominant | TT-CC | 92 (50.3) | 75 (47.5) | 1.00 | 0.62 |
|  | TC | 91 (49.7) | 83 (52.5) | 0.90 (0.59-1.38) |  |
| rs36084323 |  |  |  |  |  |
| Codominant | CC | 58 (31.7) | 41 (25.9) | 1.00 | 0.026 |
|  | CT | 83 (45.4) | 94 (59.5) | 0.64 (0.39-1.05) |  |
|  | TT | 42 (22.9) | 23 (14.6) | 1.34 (0.70-2.56) |  |
| Dominant | CC | 58 (31.7) | 41 (25.9) | 1.00 | 0.29 |
|  | CT-TT | 125 (68.3) | 117 (74) | 0.77 (0.48-1.24) |  |
| Recessive | CC-CT | 141 (77) | 135 (85.4) | 1.00 | **0.043** |
|  | TT | 42 (22.9) | 23 (14.6) | **1.79 (****1.02-3.15)** |  |
| Overdominant | CC-TT | 100 (54.6) | 64 (40.5) | 1.00 | **0.0098** |
|  | CT | 83 (45.4) | 94 (59.5) | **0.57 (0.37-0.87)** |  |
| rs1882545 |  |  |  |  |  |
| Codominant | GG | 83 (45.4) | 66 (41.8) | 1.00 | 0.32 |
|  | GA | 85 (46.5) | 71 (44.9) | 0.97 (0.62-1.53) |  |
|  | AA | 15 (8.2) | 21 (13.3) | 0.57 (0.27-1.20) |  |
| Dominant | GG | 83 (45.4) | 66 (41.8) | 1.00 | 0.56 |
|  | GA-AA | 100 (54.6) | 92 (58.2) | 0.88 (0.57-1.36) |  |
| Recessive | GG-GA | 168 (91.8) | 137 (86.7) | 1.00 | 0.13 |
|  | AA | 15 (8.2) | 21 (13.3) | 0.58 (0.29-1.18) |  |
| Overdominant | GG-AA | 98 (53.5) | 87 (55.1) | 1.00 | 0.72 |
|  | G/A | 85 (46.5) | 71 (44.9) | 1.08 (0.70-1.66) |  |
| rs870849 |  |  |  |  |  |
| Codominant | CC | 131 (71.6) | 113 (71.5) | 1.00 | 0.84 |
|  | CT | 44 (24) | 40 (25.3) | 0.97 (0.59-1.62) |  |
|  | TT | 8 (4.4) | 5 (3.2) | 1.39 (0.44-4.39) |  |
| Dominant | CC | 131 (71.6) | 113 (71.5) | 1.00 | 0.93 |
|  | CT-TT | 52 (28.4) | 45 (28.5) | 1.02 (0.63-1.65) |  |
| Recessive | CC-CT | 175 (95.6) | 153 (96.8) | 1.00 | 0.56 |
|  | TT | 8 (4.4) | 5 (3.2) | 1.40 (0.45-4.38) |  |
| Overdominant | CC-TT | 139 (76) | 118 (74.7) | 1.00 | 0.86 |
|  | CT | 44 (24) | 40 (25.3) | 0.96 (0.58-1.58) |  |

^a^Logistic regression analyses adjusted for age and gender.
